# Elimination microplastic particles in brine process water for ensuring the safety of brined cabbage

**DOI:** 10.1016/j.heliyon.2024.e25984

**Published:** 2024-02-10

**Authors:** Sora Yoon, Hyeyeon Song, Yun-Mi Dang, Ji-Hyoung Ha

**Affiliations:** Hygienic Safety Materials Research Group, World Institute of Kimchi, Gwangju 61755, South Korea

**Keywords:** Brine, Brining process, Filtration, Microplastics, Removal effect

## Abstract

Various studies have investigated the presence of microplastics (MPs) in food and their potential hazardous impact on human health. The frequency of human exposure to MPs, particularly through the consumption of manufactured food and drinking water, is increasing. However, data regarding MP contamination in brine and brined cabbage used for the production of kimchi are limited. Here, we quantified MPs in brine process water during the production of brined cabbage. Pretreatment of the brine process water by performing a filtration step resulted in an MP-removal efficiency of 98.7–100%; 3671 ± 174 MP particles were observed in brining process water that was not filtered. A glass filter, STS filter, and Si Filter showed significant MP-removal efficiency, decreasing the number of MP particles in brining process water to 2,361, 2,775, and 3,490, respectively (p < 0.05). Our results provide data on how filtering of brine can effectively safeguard kimchi from MP contamination and e can be produced. However, to overcome the limitations of our laboratory-scale study, additional technologies should be used in the future for large-scale filtration processes.

## Highlights

• Each sample investigated in the present study was contaminated with microplastics (MP).
• Fragment microplastics are more common than fibrous microplastics.
• Brine filtering and a wash process resulted in an MP removal efficiency of 98.7–100%.

## 1 Introduction

Plastic materials have continuously been used in a wide range of products since plastic was invented in 1907 [1]. Plastics are also used in the food industry, and their usage in other industries is increasing owing to their durability, weightlessness, portability, convenience, and versatility. The annual amount of plastics used worldwide has increased considerably in the recent decades. Although the number of plastic products produced globally is tremendously high, the proportion of plastic waste that is recycled is relatively low, which has resulted in widescale environmental plastic pollution that damages marine ecosystems and ultimately affects human lives [2]. To fundamentally prevent contamination by MPs, research efforts have been dedicated to the valorization of natural fibers and resins, aiming to produce biodegradable products [3]. In recent decades, microplastic (MP) debris (measuring 0.1–5000 μm in size) has been considered an environmental threat that affects a broad range of ecosystems [4]. MPs are recognized as being widespread anthropogenic pollutants, and have been observed across aquatic environmental areas, including ponds [5], estuaries [6], rivers [7], lakes [8], islands [9], and oceans [10].

MPs are derived from polyvinyl chloride, polypropylene (PP), polystyrene (PS), polyamide (PA), and polyethene (PE) according to their chemical composition and atomic structure [11], and the material characteristics of the plastic materials that result in MPs differ depending on their intended purpose [12]. The particle sizes of plastics can be divided into macroplastic (>25 mm), mesoplastic (5–25 mm), MP (1–5 mm), and nanoplastic (<1 μm) [13]. Of these, nanoplastic materials are recognized as potentially harmful to the natural environment and are continuously produced from the fragmentation of micro-to nano-scale particles [14].

Numerous environmental and marine studies have conducted quantitative assessments MPs in the environment [11,15,16]. The primary routes through which MPs enter the marine environment are via major rivers, land-based sources, and others [17]. Many studies monitoring marine products have reported on the severity of MP contamination [[18], [19], [20], [21]], and various edible marine products that are primary human food sources (such as solar sea salt, algae, and aquatic animals) are directly exposed to MPs.

Nanoplastics and MPs are recognized as new hazardous materials as they can eventually affect humans after uptake through the marine food chain and consumption of seafood and other marine-based products such as algae and solar salt [22,23]. MPs also enter the human food chain through the food manufacturing process, mostly via including soil, process water, uncontrolled agricultural products, and sea salts [[24], [25], [26]]. Several studies have evaluated the removal of MPs [[27], [28], [29], [30], [31]]. However, a study has demonstrated that such removal was insufficient [32]. Some authors reported that conventional water treatment processes could eliminate a large number of MPs; however, the effectiveness varies depending on the environmental conditions [33,34].

In recent years, the dynamic membrane technique has attracted considerable attention owing to its relatively low-energy consumption, low cost, and ease of operation [35]. This technique is advantageous because it operates under low *trans*-membrane pressure and low filtration resistance [36]. Therefore, the whole filtration process can be performed using only gravity, without pumps. Moreover, because low-density particles can be removed using the dynamic membrane, it has been recognized as a promising treatment option for fine particle removal during process water treatment [35]. Previous studies have designed, operated, and investigated the efficiency of the dynamic membrane technique in the context of MP removal under gravity-driven operation in laboratory-scale dynamic membrane filtration setups [36,37].

In the present study, we explored how the vast number of MPs introduced in the brined cabbage stage during kimchi manufacturing could be removed. Our study focused on: a) quantifying the MPs present in brine process water samples from a kimchi manufacturing factory and examining their distribution; b) analyzing the effect of a pre-filtering base in the dynamic membrane technique applied to brine process water on MP removal; and c) evaluating the effect of combined filtration and washing processes on MP removal.

## 2 Materials and methods

### 2.1 Brine monitoring and chemical agent preparation

**Fig. 1:**
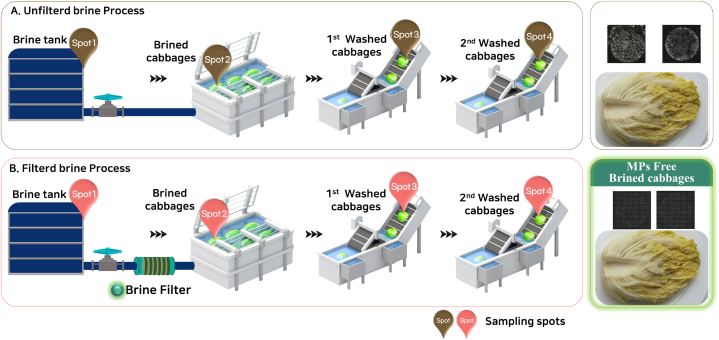
Schematic illustration of the kimchi manufacturing process for brined cabbage.

We collected brine process water samples from ten kimchi manufacturing plants located in Gwangju and Chungcung-do, Korea, from June to July 2022. A schematic flow chart of the kimchi manufacturing process is shown in the Supplementary Material, and the brine and brined cabbage spots sampled in this study are marked (Fig. 1 & Fig. S1). We collected 1 L of brine process water for MP quantification. The actual sample images, MP images, and the MP spectra map of the collected brine process water samples are shown in Fig. S2. Experiments were performed in triplicate. All chemical reagents, including hydrogen peroxide and ultrapure water, were filtered using a 5-μm stainless-steel filter mesh (TWP Inc., Berkeley, CA, USA) prior to performing the experiments. All beakers, vessels, and glassware used in the experiments were cleaned and rinsed with filtered distilled water and ethanol prior to the experiments.

### 2.2 Filtration and washing process

#### 2.2.1 Brine sampling and filtration

Brine process water (as the standard brine solution) contaminated with MPs at a level of 3671 ± 174 particles/L was obtained from kimchi manufacturing plants in Gwangju, Korea, to investigate the MP removal effect. MPs in the provided standard brine sample were quantified following the description in Section 2.3. Brine process water (i.e., the standard brine solution used in all processes) was used after continuous stirring to obtain a MP-homogenized standard brine solution. Three types of materials for filtration were examined in this study: i) a glass microfiber filter (Whatman Grade GF 10 glass microfiber filter); ii) an STS filter (20-μm stainless-steel filter mesh, Sintered 316L, Hengko Technology Co., Ltd., China); and iii) an SI filter (20-μm BioTect Ultra ceramic, Doulton Ltd., United Kingdom). For the standard brine samples filtered using each filter, MP precision analysis was performed by recovering the number of filtered MP particles.

#### 2.2.2 Experimental setup

Fig. S3 presents a schematic illustration of the laboratory-scale experimental setup for the dynamic membranes used in this study. The experimental filtration setup consisted of a brine tank containing the standard brine solution, a filtration setup, and a brine tank for storing the filtered brine solution. A brine storage tank (50-L working volume) that was large enough to perform the filtration test in batch mode was designed. A peristaltic pump was used to filter the supplying feed water (brine) from the brine tank to the storage tank containing filtered brine solution.

#### 2.2.3 Brined cabbage sampling

For each individual process, a kimchi manufacturing flow chart is shown in Figs. S1 and S2. The brined cabbage sampling spots sampled in this study are marked in Fig. 1. All brined cabbage samples were prepared using pre-filtered and unfiltered brine. Brined cabbage was collected, and the MPs remaining in this cabbage were analyzed. In addition, brined cabbage that was washed once or twice was collected and analyzed for remaining MPs. To evaluate the quantity of MPs in the cabbage samples after the completion of each process (brining, first washing, and second washing), MPs attached to the cabbage were eluted with 1 L of ultrapure purified water (approximately 400 g/each).

### 2.3 MP identification

#### 2.3.1 MP extraction

All analyses of eluate samples from each brined cabbage preparation, pretreatment, and filtration process were conducted inside Horizontal Laminar Flow Cabinets (LHG-4DS-F8, Esco Technologies, Inc., Horsham, PA, USA) to block cross-contamination from indoor airborne MPs. In addition, all test eluate samples were wrapped in aluminum foil when they were moved outside the horizontal laminar flow cabinets. To avoid cross-contamination of eluate samples, the use of plastic material was limited whenever possible, and nitrile gloves and cotton coats were employed in all processing steps. Each eluate sample was transferred to a clean 1-L glass beaker, where 200 mL of 10% potassium hydroxide (KOH) solution was added and heated to 40 °C on a heated plate for 24 h [38], after which 50 mL of 30% hydrogen peroxide (H_2_O_2_) was added over 4 d. As overflow can occur when H_2_O_2_ reacts with KOH, H_2_O_2_ was added gradually in amounts of 1–5 mL. After digestion, which took 3–5 days, the solution in the flask was vacuum filtered through a 20-μm metal filter. To remove remaining organic matter after KOH digestion, a Fenton reaction procedure was used based on a method presented previously [22]. Shortly after filtering the KOH-digested solution described above, the metal filter was transferred to a clean 1-L Erlenmeyer flask. Subsequently, 10 mL of iron sulfate heptahydrate (FeSO_4_·7H_2_O, 20 g/L) was added, followed by 20 mL of H_2_O_2_; 5 mL of hydrogen peroxide was added 1 min later, and this was repeated for the next 10 min while the flask was shaken continuously. After all H_2_O_2_ had been added, the solution was cooled to room temperature, and 4 mL of sulfuric acid (H_2_SO_4_, 98%) was added. When the solution became transparent, 10 mL of 0.1% Tween 20 solution was added to prevent MPs from adhering to the glass wall. After digestion, the solution was vacuum filtered using a silicon filter (10 × 10 mm square filters, 17-μm pore diameter). The flask was washed at least thrice with filtered ultrapure water.

#### 2.3.2 Fourier-transform infrared spectroscopy (FT-IR) measurements

The samples were measured using FT-IR spectroscopy on an FT-IR microscope (LUMOS II, Bruker Optics, Billerica, MA, USA) equipped with a 32 × 32 pixel Focal Plane Array detector. The infrared (IR) images were measured in transmission mode at a spectral resolution of 12 cm^−1^ within a spectral range from 4000 to 700 cm^−1^ and by one scan. Before IR imaging, a photograph of the sample was taken to visualize any changes in the morphology of the particle surface. Data analyses were conducted using siMPle, freeware that is capable of rapid MP material detection and that has an algorithm that compares the IR spectrum of the sample with a reference spectrum within the database, thereby allocating the material with probability scores.

### 2.4 Calculation of MP removal efficiency

MP removal efficiency was calculated using the following equation:

$$\text{RE}\left(\%\right)=\frac{\left(C_{p}-C_{n}\right)\;}{C_{p}}\times 100\text{,}$$

where *C*_*p*_ is the number of MPs collected from the previous process and *C*_*n*_ is the number of MPs collected from the next process.

## 3 Results and discussion

### 3.1 MP abundance characteristics in brine samples

**Fig. 2:**
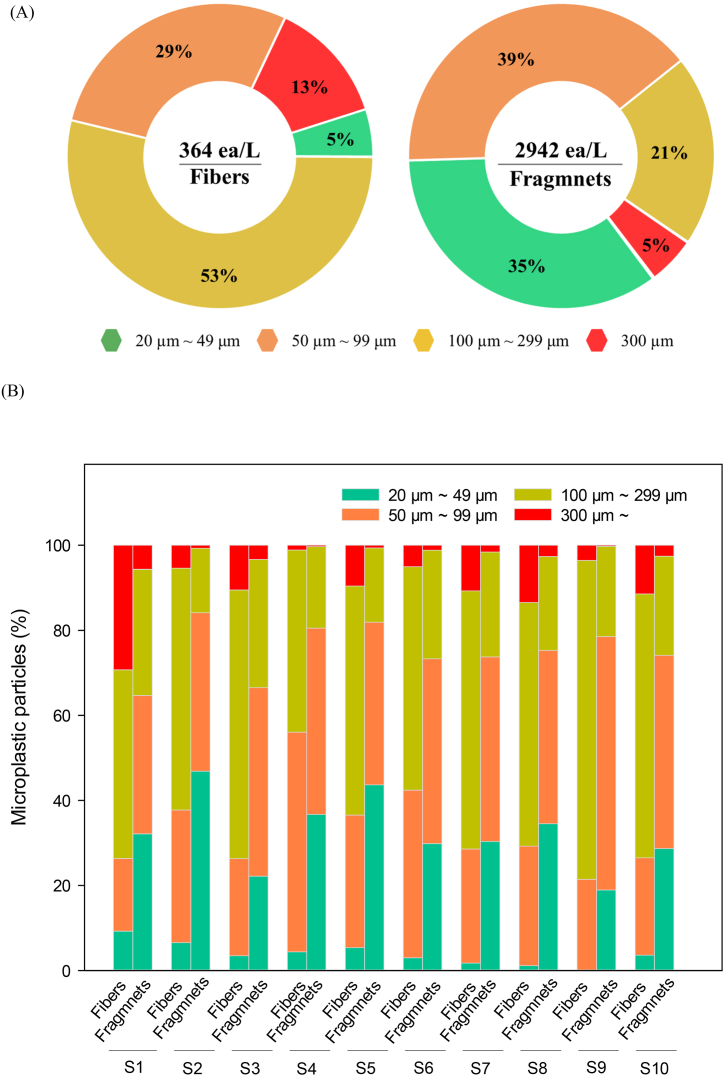
(A) Microplastic composition, size, and distribution (expressed in percentage) in brine. (B) Comparison between microplastic sizes in brine samples.

In this study, we isolated and characterized particles from ten brine process water samples. Each sample was contaminated with numerous MPs. MPs were broadly detected throughout most brine process water samples, with a mean total count of 3306 particles/L (Fig. 2). To identify the cause of MP pollution in brine process water, a complex and detailed analysis is necessary, as MP pollution in brine process water can occur through various pathways [24,26]. One such pathway is the use of sea salt in brine production. Various studies have focused on monitoring seawater and sea salt as environmental distribution indicators of MP pollution [18,39,40]. Several studies have shown that commercial solar sea salt is an acceptable indicator of the MP contamination level in seawater sources and of human exposure to MPs derived from the marine environment (unless samples are cross-contaminated during procedures) [19,41]. Detection of MPs in brine process water suggests that MPs are ubiquitous in all parts of the environment, including drinking water, air, dust, and food, and that humans can consume MPs through inhalation, ingestion, and skin contact.

### 3.2 Morphological characteristics of MPs in samples

There was a mean of 2942 particles/L of fragment MPs in the brine process water samples, and a mean of 364 particles/L of fibrous MPs; i.e., fragment MPs were much more abundant (approximately 11% fiber and 89% fragment) (Fig. 2). Similarly, Kooi and Koelmans [42] found MPs in all analyzed sea salt samples and found that the most frequent MP type was fragment particles. Seth and Shriwastav (2018) also detected MPs in all salt samples, and reported that 16 and 84% of the extracted MPs were fiber and fragment particles, respectively. This dominance of fragment MPs has also been reported in several other studies [19,43]. However, in the present study, analysis of the size of MPs based on the two shape types showed different patterns (Fig. 2A), and MPs with various sizes were observed based on the two shape types. The size distribution pattern of fiber MPs decreased in the order 100–299 μm (53%) > 50–99 μm (29%) > 300 μm (13%) > 20–49 μm (5%); the size distribution pattern of fragment MPs decreased in the order 50–99 μm (39%) > 20–49 μm (35%) > 100–299 μm (21%) > 300 μm (5%). The most common size groups were 100–299 and 50–99 μm for fiber and fragment MPs, respectively.

### 3.3 Polymer identification of MPs in samples

**Fig. 3:**
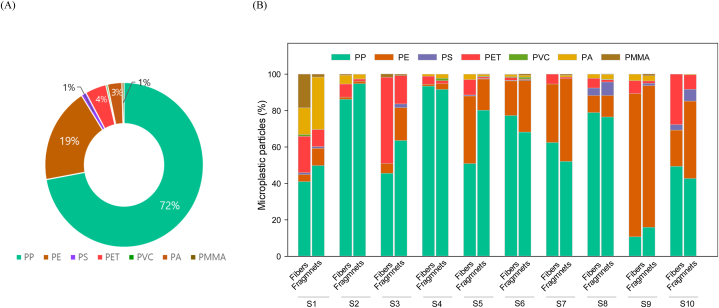
(A) Compositions and distribution (expressed in percentage) of microplastic polymer types in brine. (B) Comparison between microplastic polymer types in brine samples.

Analysis of the MP polymer composition in solar sea salts showed that the main MP types were PP, PE, polyethene terephthalate (PET), and PA, which accounted for 72, 19, 4, and 3% of the MP content, respectively (Fig. 3). In a previous study, PP, PE, and PET were dominated in sea salt samples [19]. In contrast, the entire proportion of the other polymer MP types, such as polyvinyl chloride, PS, and polymethylmethacrylate accounted for <1% of the MP content (Fig. 3). The FT-IR spectra of the sampled MPs are shown in Fig. S4. Seth and Shriwastav [44] also identified various types of MPs, including PA, polyesters, and PS in sea salt samples.

### 3.4 MP abundance characteristics in samples

Recently, public concern has increased with respect to the possible health hazards of MPs. Although the removal of colloids and solids is specific to drinking water production, drinking water treatment plants are not specifically required to eliminate MP particles [30]. Furthermore, there is no obligation regarding a limit for MPs in brine process water. While scientific evidence unequivocally establishes the presence of MPs in various food products, a notable gap exists in understanding the fate of MPs in the human body after their ingestion [45]. Consequently, the current knowledge in this domain remains highly limited; information regarding the effects of MP ingestion on human health [46] and the associated risks [47] is scarce. As a result, comprehensive risk assessment studies involving MPs are difficult to perform.

We therefore focused on MP removal using prefiltration processing steps. Differences in the MP count in brine water and each treatment stage (brining, first and second wash processes) are represented as removal efficiency (%) in the present study. Removal efficiency was analyzed to determine the MP removal efficiency of the prefiltration processing steps. The mean MP removal efficiency by a single brine process was 27.6%, with 2658 MPs transferred from brine process water to brined cabbage (Table 1). By evaluating the additional removal efficiency through the first and second wash processes, we found the removal efficiency to be 41.7% (first wash) and 54.1% (second wash). In the absence of prefiltration of brine process water, 2658, 2142, and 1686 MPs were transferred to brined cabbage, cabbage washed once, and cabbage washed twice, respectively, and were not completely removed by either the brine process or the first and second wash processes. When using the filtered brine, the removal efficiency was 98.7–100% after the three processes (brining process, first and second wash process) had been performed consecutively (Table 1). In particular, the effect of using the SI filter was the best, and MPs were not detected in the brined cabbage that went through the first washing process.

**Table 1:** Microplastic quantification and removal efficiency in brining process water, brined cabbage, and washes brined cabbage samples by individual process.

Filtration	Brining process water		Brined cabbages		Number of washes Brined cabbages			
					1st washed		2nd washed	
	Particles (ea/L)	Removal efficiency (%)	Particles (ea/kg)	Removal efficiency (%)	Particles (ea/kg)	Removal efficiency (%)	Particles (ea/kg)	Removal efficiency (%)
No-filter Filtration	3671 ± 174^A,a^	–	2658 ± 313^A,b^	27.6	2142 ± 152^A,b^	41.7	1686 ± 108^A,c^	54.1
Glass filter	1310 ± 58^B,a^	64.3	905 ± 42^B,b^	74.1	162 ± 34^B,c^	95.6	46 ± 16^B,d^	98.7
STS Filter	896 ± 23^C,a^	75.6	570 ± 27^C,b^	84.5	84 ± 8^C,c^	97.7	ND	100
Si Filter	181 ± 22^D,a^	95.1	98 ± 24^D,b^	97.3	ND^a^^)^	100	ND	100

A–D: letters within the same column indicate significant (p < 0.05) differences. a–d: letters within the same row indicate significant (p < 0.05) differences.

The concentration of MPs in the initial brining process water is 3671 ± 174 particles per liter.

a ^)^ND indicates not detected.

We tested the hypothesis that MP particles originating from saline water are transferred to food items, such as brined cabbage. Previous findings from our research confirmed the efficacy of the first stage of the filtration process in removing MPs that could potentially be transferred to food. Despite the escalating concerns regarding the contamination of food with MPs from seawater or sea salt, data regarding the relationship between MPs in brine and those present in brined cabbage are lacking. Notably, efforts have been made to prevent the cross-contamination of secondary processed products by adequately removing MPs from brining process water samples. For example, Gonźalez-Camejo et al. [47] employed a four-step sequential filtration method, utilizing membranes with pore sizes of 0.1, 0.45, 2.5, and 25 μm, for effectively removing MPs of various sizes. Another study applied hydrostatic pressure to a nanoporous membrane for ultrafiltration, achieving successful MP removal [48]. Mintenig et al. [49] also used a crossflow ultrafiltration system to achieve an 88% efficiency for the removal of MPs from drinking water [46].

Various industrial fields are introducing process water treatment plant systems as a final step to prevent MPs in processing water from being discharged into the environment [30]. However, there is a lack of scientific evidence to preemptively solve the risk of MPs being transferred to manufactured food. In particular, in the food sector, in which a large amount of processed water is used, it is necessary to establish a device that can identify the transfer route of MPs to food and preemptively block this route. Although unrelated to the food sector, several studies have studied the physical removal of MPs through various pretreatment techniques such as rapid sand filtration and membrane systems [48]. To promote MP removal, we quantified MPs in brine and in filtered brine, in brined cabbage that underwent the brining process, and in brined cabbage that underwent a washing process.

## 4 Conclusion

A comprehensive evaluation of the safety associated with the removal of MPs cannot be performed currently because of the lack of extended studies. As a result, this study could only confirm that potential risks caused by MPs can be prevented by sufficiently removing MPs contained in brined cabbage. We demonstrated that incorporating the initial and subsequent washing processes, along with filtration employing an SI filter in the brining process, achieved an MP removal efficiency of 98.7–100%. However, to overcome the limitations of our laboratory-scale research, additional technologies should be used in the future for large-scale applications to expand the filtration process. Further research is warranted to develop a sustainable filter system capable of consistently maintaining high MP-removal efficiency after prolonged use.

## CRediT authorship contribution statement

**Sora Yoon:** Supervision, Software, Methodology, Investigation, Formal analysis, Data curation, Conceptualization. **Hyeyeon Song:** Validation, Software, Resources, Methodology, Investigation, Formal analysis, Data curation, Conceptualization. **Yun-Mi Dang:** Formal analysis, Data curation, Conceptualization. **Ji-Hyoung Ha:** Writing – review & editing, Writing – original draft, Investigation, Funding acquisition, Data curation.

## Declaration of competing interest

The authors declare that they have no known competing financial interests or personal relationships that could have appeared to influence the work reported in this paper.
