# Supplementary material for: Elimination microplastic particles in brine process water for ensuring the safety of brined cabbage
Source: Heliyon. 2024 Feb 10;10(4):e25984. doi: 10.1016/j.heliyon.2024.e25984 (PMC10881328; doi:10.1016/j.heliyon.2024.e25984)
Supplement: Multimedia component 1 [file mmc1.docx]

**Supplementary material**

**Elimination microplastic particles in brine process water for ensuring the safety of brined cabbage**

Sora Yoon^†^, Hyeyeon Song^†^, Yun-Mi Dang, Ji-Hyoung Ha*

*Hygienic Safety Materials Research Group, World Institute of Kimchi, Gwangju 61755, Korea*

*Corresponding author: Ji-Hyoung Ha,

Hygienic Safety Materials Research Group

World Institute of Kimchi, 86 Kimchi-ro, Nam-gu, Gwangju 61755, Korea

E-mail: [hajee@wikim.re.kr](mailto:hajee@wikim.re.kr); Tel.: 82-62-610-1845; Fax.: 82-62-610-1810

^†^The first two authors contributed equally to this study and are considered co-first authors.

**Fig. S1.** Detailed description of brine production, brining process using brine, and washing process to remove brine.

| Brine tank → | Brined cabbages → | 1^st^ Washed cabbages → | 2^nd^ Washed cabbages |
| --- | --- | --- | --- |
| 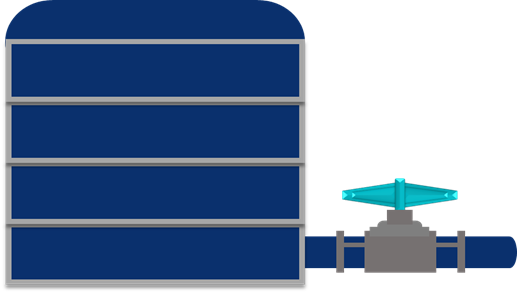Unfiltered brine  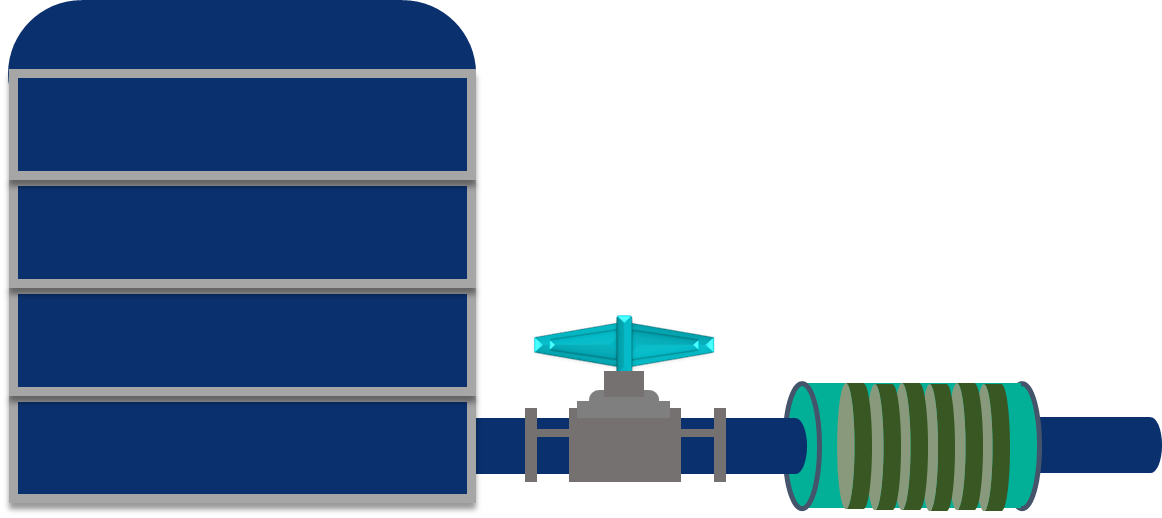  Filtered brine | 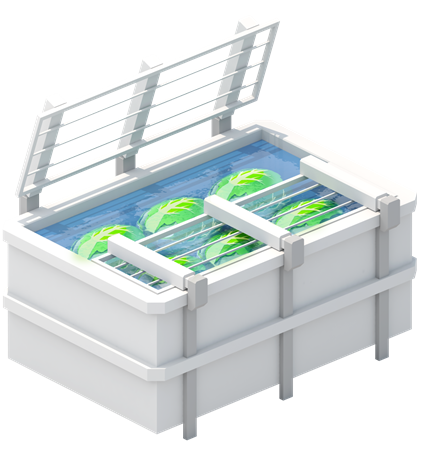 | 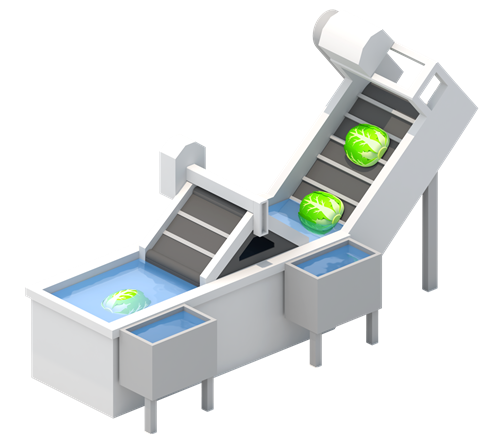 | 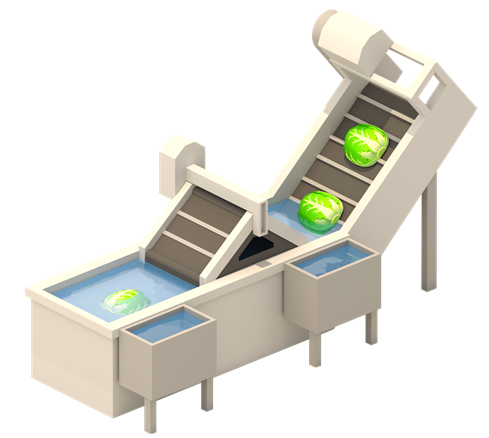 |
| In order to pickle cabbage in brine, about 10-12% high-concentration brine is produced in large quantities. | During the brining process of Kimchi cabbage, osmotic pressure between salt and Kimchi cabbage inhibits the growth of microorganisms and creates an environment so lactic acid bacteria (LAB) that withstand high salt concentrations can dominate the fermentation in Kimchi. | In order to remove brine from brined cabbage, it goes through an air bubble device-based washing process (1^st^). | In order to obtain sufficient brine removal effect, it undergoes an additional air bubble device-based washing process (2^nd^). |

**Fig. S2.** Actual sample image, MPs image, and MPs spectra map of brine process water samples collected from Kimchi manufacturing plants**.**

| Sampling sites | Sea water | | |
| --- | --- | --- | --- |
|  | Samples | Micro-particles image | Microplastic  spectra map |
| S1 | 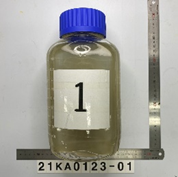 | 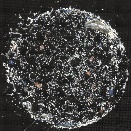 | 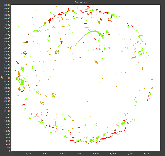 |
| S2 | 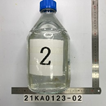 | 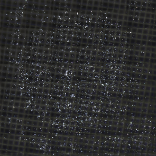 | 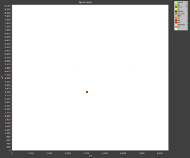 |
| S3 | 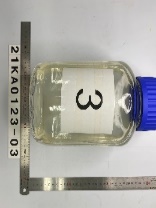 | 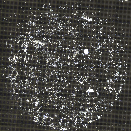 | 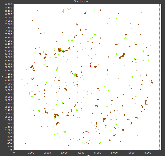 |
| S4 | 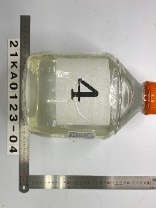 | 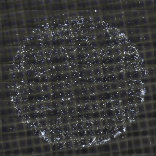 | 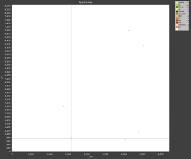 |
| S5 | 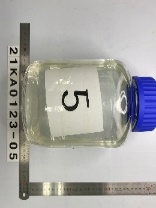 | 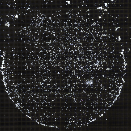 | 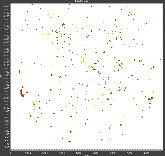 |
| S6 | 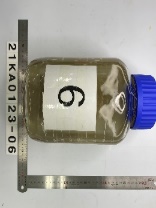 | 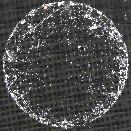 | 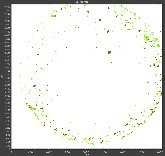 |
| S7 | 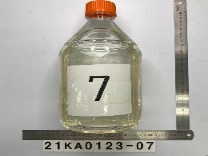 | 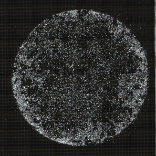 | 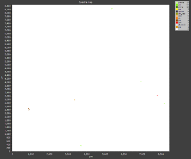 |
| S8 | 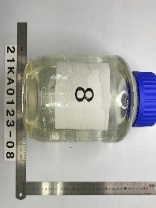 | 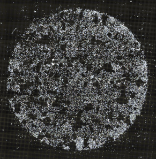 | 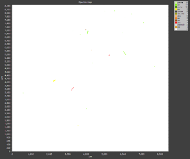 |
| S9 | 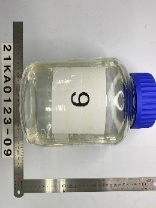 | 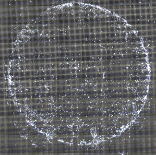 | 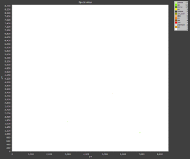 |
| S10 | 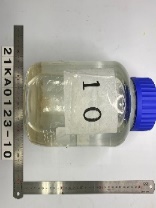 | 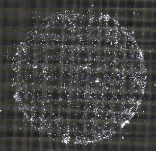 | 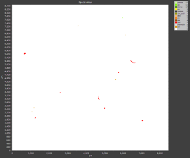 |

**Fig. S3.** Schematic illustration of laboratory-scale experimental setup for dynamic membranes.


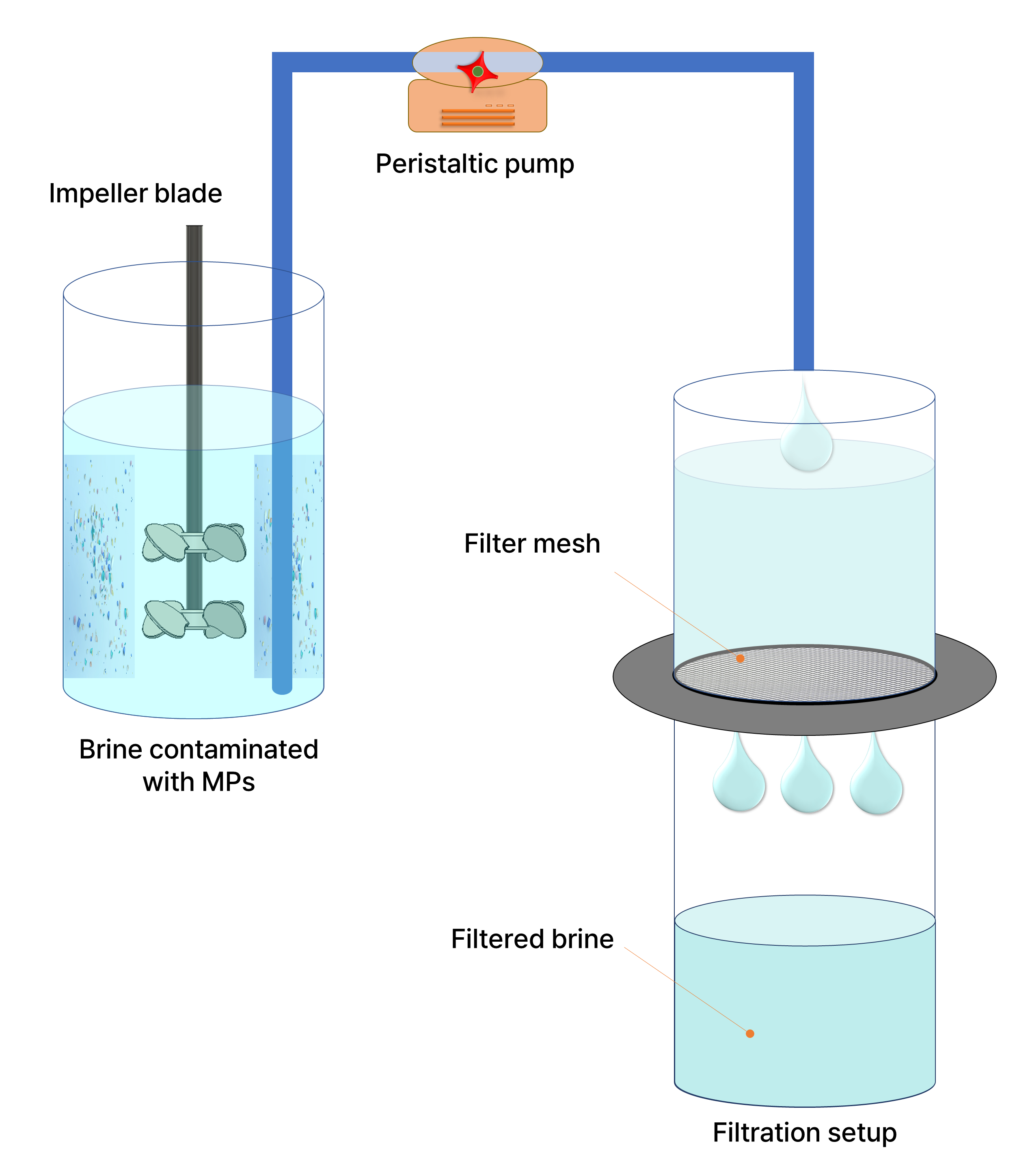


**Fig. S4.** The FTIR spectra of the collected microplastics.

| 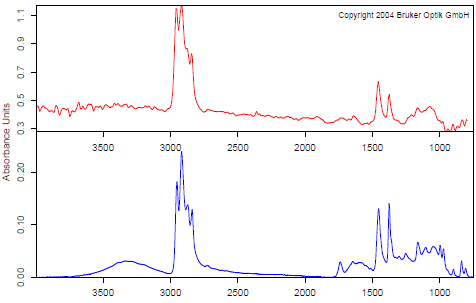 | 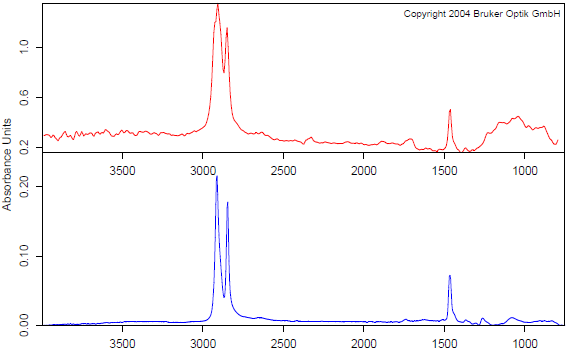 |
| --- | --- |
| PP | PE |
| 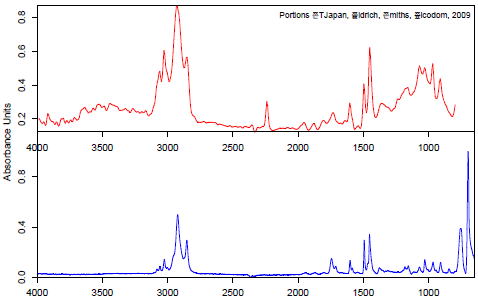 | 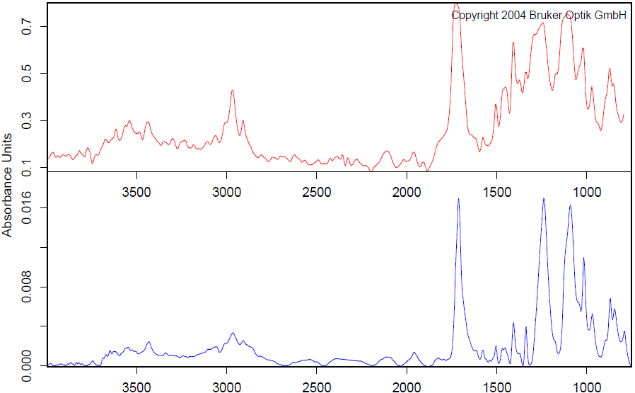 |
| PS | PET |
| 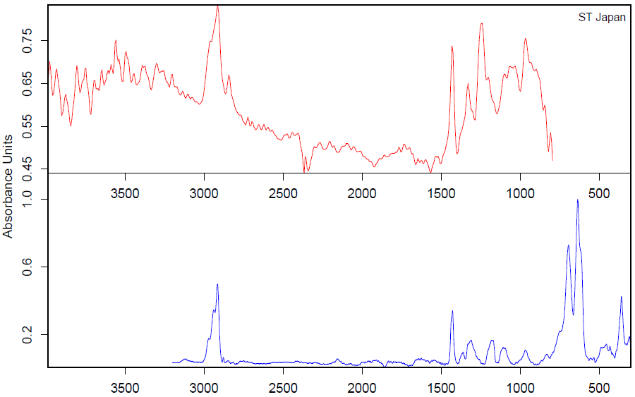 | 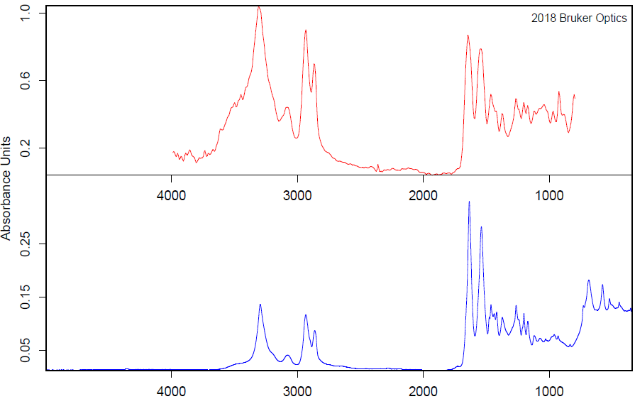 |
| PVC | PA |
| 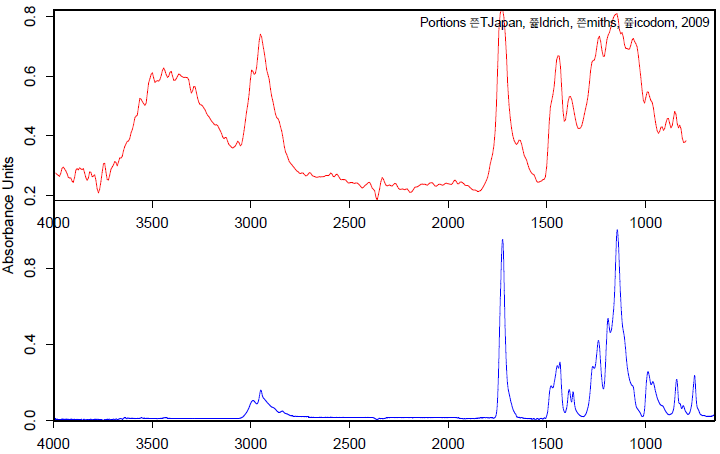 |  |
| PMMA |  |
